# Duodenal Lymphocytosis and B1 Deficiency: Unveiling the Overlap Between Gut and Brain

**DOI:** 10.7759/cureus.102917

**Published:** 2026-02-03

**Authors:** Wafa Hrouch, Yahya Naji, Lagtarna Hamza, Chaima Redouane, Achref Miry, Loubna Chouaf, Sara Laadami, Nawal Adali

**Affiliations:** 1 Department of Neurology, University Hospital of Agadir, Agadir, MAR; 2 Neurosciences Innovation Cognition Ethique (NICE), Rein, Endocrinology, Gastroenterology, Neurosciences, Ethique (REGNE) Research Laboratory, Agadir, MAR; 3 Department of Biopathology, University Hospital of Agadir, Agadir, MAR

**Keywords:** dry beriberi, duodenal lymphocytosis, gayet-wernicke encephalopathy, gut-brain connection, thiamine

## Abstract

Thiamine (vitamin B1) deficiency is a reversible, yet potentially fatal condition that affects both the central and peripheral nervous systems. Although commonly linked to chronic alcoholism, non-alcoholic causes, including malabsorption syndromes, are increasingly being recognized.

We report the case of a 22-year-old woman who presented with progressive lower limb weakness, gait disturbances, and confusional symptoms, following a history of intermittent diarrhea and vomiting. Neurological examination revealed ascending motor deficits, areflexia, cerebellar syndrome, and internuclear ophthalmoplegia. Brain magnetic resonance imaging (MRI) showed bilateral symmetrical lesions in the caudate nuclei and periaqueductal area, suggesting Gayet-Wernicke encephalopathy (GWE). The electroneuromyography (ENMG) revealed axonal sensorimotor polyneuropathy. Cerebrospinal fluid (CSF) analysis showed mild hyperproteinorachia mimicking acute inflammatory demyelinating polyneuropathy (AIDP). A profound thiamine deficiency (22.4 nmol/L) was identified alongside duodenal lymphocytosis without villous atrophy or celiac-specific antibodies. High-dose intravenous (IV) thiamine therapy led to rapid improvement in neuropsychiatric symptoms, with partial motor recovery over two months and near-complete resolution on follow-up MRI at six months.

This case highlights the diagnostic complexity of non-alcoholic thiamine deficiency, in which Gayet-Wernicke encephalopathy and dry beriberi may present atypically. MRI findings and clinical response to thiamine are key to early diagnosis. Duodenal lymphocytosis may suggest an underlying malabsorption process even in the absence of definitive celiac disease. Clinicians must maintain a high index of suspicion for thiamine deficiency in patients with neurological and gastrointestinal (GI) symptoms regardless of alcohol use. Prompt thiamine supplementation is crucial to prevent irreversible neurological damage.

## Introduction

Thiamine is a water-soluble vitamin found in various grains, nuts, and legumes. However, storage of the human body lasts for up to 18 days. After crossing the blood-brain barrier, vitamin B1 is converted to thiamine pyrophosphate (TPP) [1,2], which acts as a cofactor for several enzymes, including the α-ketoglutarate dehydrogenase complex, pyruvate dehydrogenase complex in the Krebs cycle, and transketolase in the pentose phosphate pathway [2-4]. The accumulation of by-products such as lactate, pyruvate, alanine, and glutamate leads to metabolic acidosis with focal lactic acidosis and an electrolyte imbalance that causes cytotoxic edema. Dysfunction of the blood-brain barrier results in vasogenic edema, whereas the production of free radicals leads to cell death via necrosis and apoptosis [2,3]. By day 14, brain lesions generally appear to affect metabolically active regions [5]. Pathological examination of the acute lesions showed vascular congestion with or without petechial hemorrhage, intra- and extracellular edema with swelling of astrocytes and oligodendrocytes, and varying degrees of necrosis. Chronic lesions may be demyelinating and gliotic, with brain atrophy [1,6].

Thiamine (vitamin B1) deficiency affects both the central and peripheral nervous systems, leading to various manifestations such as Gayet-Wernicke encephalopathy (GWE), a rare but life-threatening neurological emergency, and dry beriberi with peripheral neuropathy [1,7]. Although this deficiency is most commonly associated with chronic alcoholism, it can also occur outside of this addiction, with many other causes reported as predisposing factors [5]. GWE is a serious condition with a mortality rate of >30% [3]. Its prevalence can reach 12.5% in alcoholics and up to 59% in postmortem alcohol-related cases [1]. Dry beriberi appears with a gradual, insidious onset but can occasionally appear rapidly, resembling acute inflammatory demyelinating polyneuropathy (AIDP) [7,8].

Here, we present a complex clinical case of a young patient with neurological and gastrointestinal (GI) symptoms. A severe deficiency in vitamin B1 with duodenal lymphocytosis was identified, strongly suggesting a diagnosis of GWE associated with dry beriberi due to intraepithelial lymphocytosis, without a definitive diagnosis.

## Case presentation

**Figure 1:**
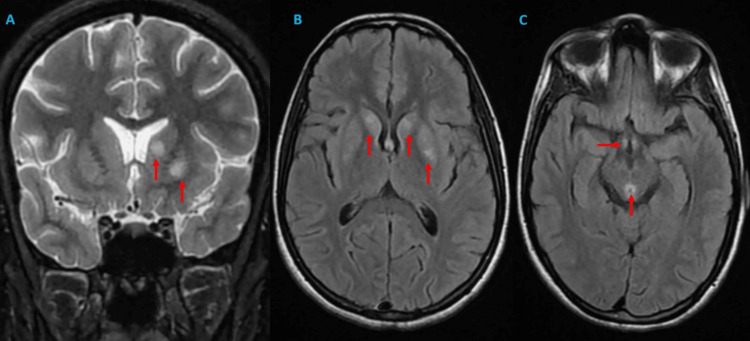
A and B: Coronal T2-weighted and axial FLAIR MRI images revealed bilateral hyperintensities (red arrows) in the caudate nuclei, along with an atypical hyperintense lesion in the left lenticular nucleus (putamen). Such basal ganglia involvement is uncommon but has been documented in non-alcoholic cases of Wernicke encephalopathy. C: Axial FLAIR MRI image demonstrates symmetric hyperintensities in the mammillary bodies and periaqueductal gray matter (red arrows), consistent with classic manifestations of thiamine deficiency. MRI: magnetic resonance imaging, FLAIR: fluid-attenuated inversion recovery

A 22-year-old woman with no significant past medical history presented with a 43-day history of intractable diarrhea and vomiting, followed by gait instability and balance disorders 10 days before admission; these rapidly progressed to symmetrical, synchronous distal sensorimotor deficits in both lower limbs with an ascending pattern. During hospitalization, the clinical picture became complicated by vertigo associated with memory and attention disorders in an apyretic context with general deterioration. On admission (hospital day 1), her weight was 40 kg (body mass index (BMI): 15 kg/m²). Progression is marked by the onset of confusional syndrome with significant temporal-spatial disorientation and visual hallucinations. Clinical examination revealed a confused patient with a Glasgow Coma Scale score of 13/15 (eyes (E): 4, verbal (V): 3, motor (M): 6), tachycardia at 130 bpm, normotension at 120/90 mmHg, polypnea at 20 breaths per minute, normal oxygen saturation at 98% in room air, and afebrile state at 37.3°C. Neurological examination showed proximal and distal muscular weakness with a Medical Research Council scale of 2/5 in the four limbs. The deep tendon reflexes were abolished with bilateral areflexia of the patellar, bicipital, and tricipital reflexes. Sensory deficits were not observed. Both static and kinetic cerebellar syndromes are associated with internuclear ophthalmoplegia. Ophthalmological examination revealed bilateral visual acuity reduction (20/40 in both eyes) with bilateral stage II papilledema on fundoscopy. Brain magnetic resonance imaging (MRI) revealed bilateral and symmetrical T2 fluid-attenuated inversion recovery (FLAIR) hyperintensities in the heads of the caudate nuclei, along with an atypical hyperintense lesion in the left lenticular nucleus (putamen), as well as involvement of mammillary bodies and periaqueductal gray matter. These findings are consistent with the classic features of thiamine deficiency, with no evidence of a tumorlike profile on spectroscopy (Figure 1).

**Figure 2:**
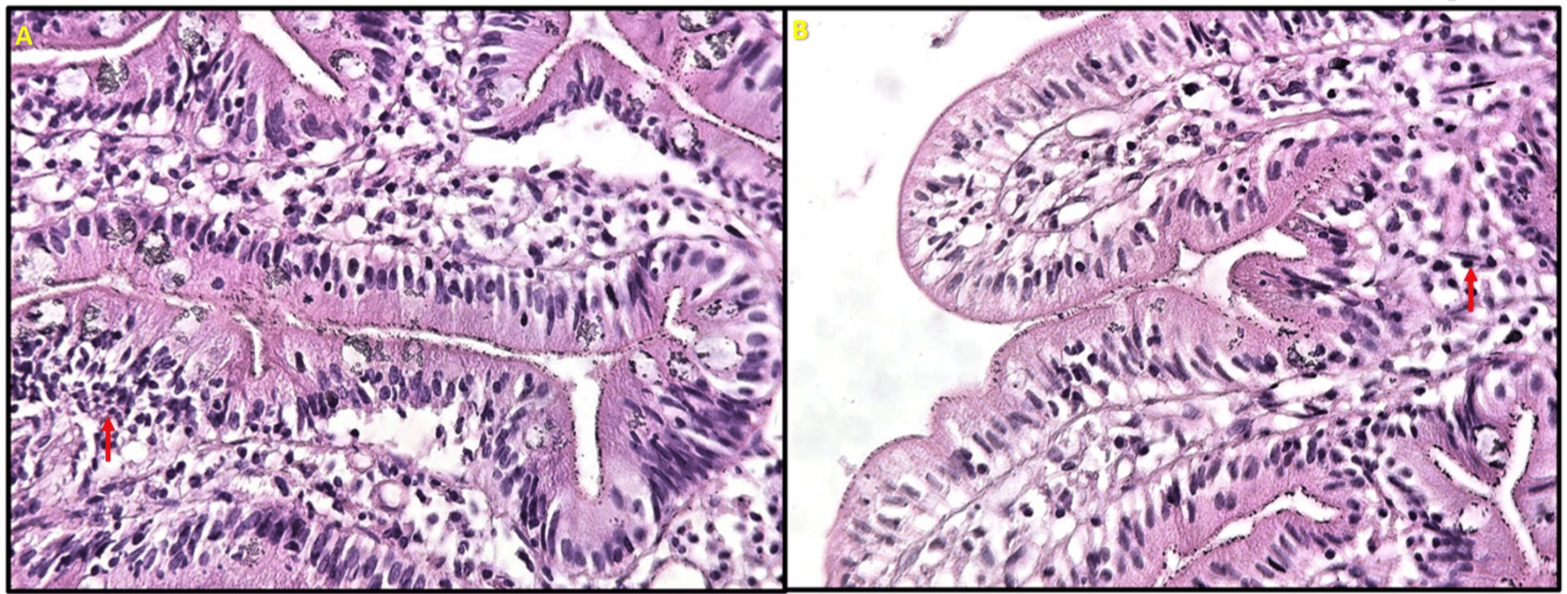
A and B: Microscopic examination of the gastroduodenal biopsy showing significant intraepithelial lymphocytosis (red arrows) in the proximal small intestine with preserved villous architecture and no evidence of pathogens or malignancy.

The electroneuromyography (ENMG) was suggestive of severe axonal sensorimotor polyneuropathy (Table 1). Lumbar puncture revealed normal opening pressure, slight hyperproteinorachia (0.6 g/L), and a normal cell count. HIV, syphilis, and Borrelia serology tests were negative. Immunological workup, including antinuclear antibodies (ANA), anti-myelin oligodendrocyte glycoprotein (MOG), anti-aquaporin-4 (AQP4), antineutrophil cytoplasmic antibodies (ANCA), and antiphospholipid antibody panels, showed no abnormalities. A chest, abdomen, and pelvis (TAP) computed tomography (CT) scan showed significant gastroduodenal dilation without identifiable obstruction. The esophagogastroduodenoscopy (EGD) revealed erythematous mucosa. Gastroduodenal biopsy revealed significant proximal small intestinal intraepithelial lymphocytosis with normal villus architecture, without pathogens or malignant cells (Figure 2). Microscopic examination of stool samples revealed no evidence of bacterial or parasitic infections. Tissue transglutaminase anti-immunoglobulin A (IgA) antibody level was negative. Whole blood vitamin B1 was profoundly low at 22.4 nmol/L (normal range: 66.5-200 nmol/L), confirming severe deficiency. The rest of the biological workup revealed iron deficiency anemia, moderate hepatic cytolysis, and hypokalemia. A cardiovascular evaluation revealed no abnormalities.

**Table 1:** Nerve conduction studies demonstrate absent or severely reduced CMAPs and SNAPs in the lower limbs, with relatively preserved amplitudes in the upper limbs. These findings are consistent with a severe axonal sensorimotor polyneuropathy. CMAP: compound muscle action potential, SNAP: sensory nerve action potential, MNCV: motor nerve conduction velocity, SNCV: sensory nerve conduction velocity

MNCV data			
Nerve	Latency	Amplitude	Velocity
Left ulnaris			
Wrist	3.39	3.5	-
Elbow	8.45	3.2	47
Right medianus			
Wrist	4.32	2.2	-
Elbow	10.57	0.5	37
Right tibial nerve	NR	NR	-
Left tibial nerve	NR	NR	-
Left peroneus	NR	NR	-
SNCV data			
Left ulnaris	NR	NR	NR
Right medianus			
Palm	NR	NR	NR
3^rd^ finger	1.61	4.5	50
Right superficial fibular nerve	NR	NR	-
Left suralis	NR	NR	-

**Figure 3:**
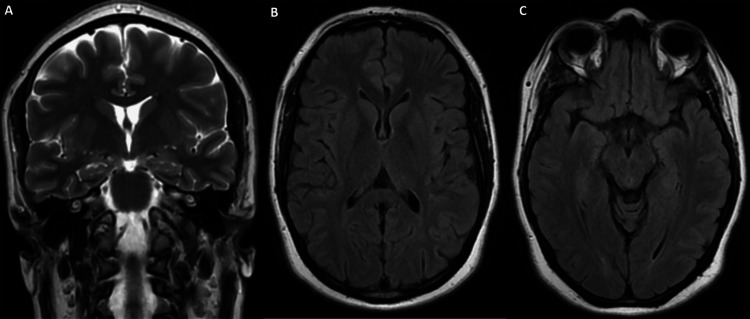
A: Coronal T2-weighted brain MRI showing disappearance of the lesion in the basal ganglia. B and C: Axial FLAIR brain MRI showing complete disappearance of lesions in the mammillary bodies and periaqueductal gray matter. MRI: magnetic resonance imaging, FLAIR: fluid-attenuated inversion recovery

Parenteral vitamin B1 supplementation was initiated at a dose of 500 mg three times daily for five days and then was switched to oral administration at 500 mg per day, accompanied by nutritional rehabilitation, antiemetics, gastric protection, and physiotherapy sessions. The short-term outcomes showed a regression of confusion. Two months later, there was a significant improvement in cerebellar syndrome and ophthalmological findings. However, a slight motor deficit persisted in both lower limbs. Follow-up MRI performed after six months showed nearly complete regression of the signal abnormalities (Figure 3).

## Discussion

Thiamine (vitamin B1) is a water-soluble vitamin that plays an important role in energy metabolism and proper functioning of several organ systems, such as the nervous, musculoskeletal, and cardiovascular systems [4]. Thiamine deficiency and impairment of these enzymes lead to a decrease in adenosine triphosphate (ATP) production and the synthesis of DNA/RNA and NADPH, impairing the resistance of cells to oxidative stress [4,9]. Lack of vitamin B1 causes GWE and dry beriberi, and non-alcoholic etiologies, although rare, have already been reported in patients suffering from malnutrition [7], hyperemesis gravidarum [4], and bariatric surgery [10], as well as in those receiving prolonged parenteral nutrition [11], in cases of tumors [12], thyrotoxicosis [13], psychiatric disorders such as anorexia nervosa [14], schizophrenia [15], inappropriate refeeding syndrome [16], and malabsorption or diarrhea [17].

The diagnosis of GWE is primarily clinical, based on the "classic triad," which includes a change in mental status [7,9], due to involvement of the thalamic nuclei or mammillary bodies [18], followed by oculomotor disturbances (nystagmus, and third and sixth cranial nerve palsies) [7,9], and gait ataxia associated with a central vestibular syndrome and a cerebellar syndrome. This triad is only observed in 16% of patients [1,3,9] and may present with hypothermia or hyperthermia [3], hypotension and/or tachycardia [18], hypoacusis or deafness [16], decreased visual acuity [19], seizures related to glutamatergic hyperactivity [18], dysarthria [9], and abnormal movements, and psychiatric disturbances such as psychosis with hallucinations and delirium, or bulimia have also been described [20]. Its incidence in non-alcoholics is 0.8%-2.8% in autopsy reports and only 0.04%-0.13% in clinically reported cases. It is often undiagnosed in non-alcoholic patients because of a low level of suspicion, which is linked to misleading clinical forms and atypical presentations in patients not recognized as being at risk [5].

Brain MRI remains the best method to confirm diagnosis [18], with a sensitivity and specificity of 53% and 93%, respectively [1,7]. The most distinctive abnormalities are cytotoxic and vasogenic edema in the form of hyperintensities on T2-weighted, FLAIR, and diffusion MRI, typical of their locations and symmetrical characteristics, notably at the level of the medial thalami, particularly along the wall of the third ventricle (80%-85%), periaqueductal area (59%-65%), mammillary bodies (38%-45%), and tectal plate (36%-38%) [18]. These lesions were inconsistently enhanced after gadolinium injection. Diffusion sequences identify hyperintense areas that predict long-term neurological sequelae [20]. Atypical locations have been reported, including the corpus callosum, brainstem, cerebellum, head of the caudate nuclei, lenticular nuclei, red nuclei, vestibular nuclei, and frontal and parietal cortex [1,20,21]. The bilateral caudate head and left putamen T2 FLAIR hyperintensities represent an atypical MRI pattern in non-alcoholic Wernicke encephalopathy (WE), seen in 10%-20% of severe malnutrition cases versus classic periventricular/mammillary involvement (80%-85%). Differentials for symmetric basal ganglia lesions include Leigh syndrome (excluded by acute GI onset, no maternal inheritance), hypoxic-ischemic injury (no cardiorespiratory arrest, complete reversal post-thiamine), Wilson's disease (normal liver enzyme and ophthalmic assessment), carbon monoxide poisoning (no exposure, caudate lesion more than globus pallidus), methanol toxicity (no exposure), infectious disease Enterovirus D68, West Nile, and Lyme (negative viral/cerebrospinal fluid (CSF) polymerase chain reaction (PCR) panel, no fever/rash), and deep cerebral venous thrombosis (normal venous imaging, non-hemorrhagic). Proton MRS leads to GWE with preserved NAA/Cr (intact neurons) and isolated lactate elevation (metabolic stress) rather than neoplastic profile (choline↑/lipid doublet), while dramatic clinical/MRI resolution within three months, driven by early thiamine repletion despite extreme catabolism (40 kg), distinguished this reversible emergency from progressive mimics [18].

While dry beriberi manifests as symmetric peripheral neuropathy with ataxia, areflexia, and painful sensorimotor deficits that may mimic Guillain-Barré syndrome (GBS), occurring in 11% of thiamine deficiency cases, the wet form predominantly involves cardiovascular dysfunction, presenting with high-output heart failure, tachycardia, peripheral edema, and dyspnea from thiamine-dependent vasodilation and myocardial impairment [7,21]. Its severity correlates with the degree and duration of thiamine deficiency and may be associated with GWE and Korsakoff syndrome [2]. Nerve conduction studies typically show predominant sensory axonal neuropathy [22]. Nerve biopsies primarily show axonal degeneration and progression to demyelination during the terminal phase of the disease [7]. Another factor that can complicate diagnosis is the CSF profile, which may show mild hyperproteinorachia that mimics the albuminocytological dissociation commonly observed in GBS. The mechanism of albuminocytological dissociation has not been fully elucidated [7,21]. In the literature, acute beriberi neuropathy mimicking Guillain-Barré syndrome (GBS) is rarely reported and should be considered in anyone at risk of thiamine deficiency [22]. While treatment with intravenous (IV) human immunoglobulin may take 2-4 weeks to show an effect on GBS treatment, patients with beriberi generally demonstrate a more rapid clinical improvement after thiamine supplementation [7]. Our patient presented with ascending weakness in the lower limbs, with cerebrospinal fluid (CSF) results compatible with Guillain-Barré syndrome (GBS). However, the onset of confusion and oculomotor disturbances led to the diagnosis of GWE associated with dry beriberi. Despite atypical MRI findings and a misleading initial presentation, the diagnosis was made based on a combination of factors and after excluding all differential diagnoses, in addition to the thiamine level measurement that confirmed B1 deficiency. Thiamine deficiency results from malabsorption syndrome, caused by intermittent diarrhea and vomiting, along with duodenal lymphocytosis.

Duodenal lymphocytosis is characterized by the presence of lymphocytes in the epithelial layer of the duodenum while maintaining a normal villous architecture. These lymphocytes are called "intraepithelial lymphocytes" (IELs) and are T cells that express CD3 on their surfaces [23]. Their exact significance in duodenal biopsies remains poorly understood and continues to be a subject of inquiry for pathologists and clinicians. IELs can be observed in the normal duodenal mucosa in up to 3% of duodenal biopsies [24], where they are an integral part of the host immune system and play essential roles in immune surveillance and activation in response to dietary antigens [23]. The main causes of duodenal lymphocytosis include gluten-related disorders, non-gluten food hypersensitivity, and *Helicobacter pylori* infection [24,25]. Regarding negative etiological screening, we assumed that it could be idiopathic, including irritable bowel syndrome. However, patients with less destructive histological lesions may be negative for anti-tissue transglutaminase IgA antibodies without excluding the diagnosis of celiac disease [24].

GWE and dry beriberi are reversible medical emergencies, and any delay in treatment leads to permanent neurological damage or even death [18]. Treatment relies on parenteral thiamine therapy, which is indicated even in suspected cases before a definitive diagnosis is made [9]. Various protocols have been proposed to achieve this purpose. The lack of randomized controlled trials has prevented the establishment of evidence-based guidelines for the administration of thiamine. To date, there is no consensus on the optimal dose, treatment duration, or method of thiamine administration [18]. According to the Royal College of Physicians and the European Federation of Neurological Associations, administration of a high dose of thiamine occurs immediately via the intravenous (IV) or intramuscular (IM) route [9]. The recommended dosage is 500 mg IV or IM three times a day for 2-3 days, followed by 250 mg IV or IM three times a day for the next 3-5 days [4,9,21], followed by oral thiamine 100 mg three times a day for the duration of hospital stay and outpatient treatment [21]. The IV infusion should be slow, over 30 minutes, diluted in 100 mL of normal saline to avoid hypersensitivity reactions to thiamine [3,9], although this risk remains extremely low [18]. Anaphylactic shock has been reported in four cases per million IV administrations and in one case per five million IM administrations [2]. The exact duration for thiamine supplementation is not clearly defined in guidelines; however, treatment should be continued until all residual deficits resolve clinically, with serial monitoring of symptoms rather than levels alone. Repeat plasmatic B1 testing should be done 1-2 weeks after initiating or adjusting supplementation to evaluate response, ensuring correlation with clinical status [18,21]. The prognosis depends on the stage of the disease and the rapid initiation of thiamine treatment. If treated quickly and adequately, it allows for total reversibility of the symptoms [9]. Initial symptom improvement can be observed within the first week, but it generally takes 1-3 months to fully resolve [18].

## Conclusions

Gayet-Wernicke encephalopathy (GWE) and dry beriberi remain underdiagnosed medical emergencies that can lead to irreversible neurological damage or death if not promptly recognized and treated. Diagnosis is challenging, particularly in non-alcoholic patients, due to atypical presentations and the need for immediate intravenous thiamine therapy, which is safe and highly effective. A high index of suspicion is essential, as the classic triad is observed in only one-third of the cases. Diagnosis relies on the clinical context, neuroimaging, and, crucially, the patient's response to thiamine supplementation.

This case report highlights a rare presentation of dry beriberi mimicking AIDP and GWE in a young woman with gastrointestinal symptoms and duodenal lymphocytosis. This underscores the importance of considering thiamine deficiency in patients with unexplained neurological symptoms and potential for malabsorption. Early recognition and treatment remain the cornerstones of favorable outcomes.
